# Enhancedanti-tumor efficacy through a combination of intramuscularly expressed DNA vaccine and plasmid-encoded PD-1 antibody

**DOI:** 10.3389/fimmu.2023.1169850

**Published:** 2023-04-17

**Authors:** Xun Liu, Yueyao Yang, Xiufeng Zheng, Ming Liu, Gang Wang

**Affiliations:** ^1^ National Engineering Research Center for Biomaterials, College of Biomedical Engineering, Sichuan University, Chengdu, Sichuan, China; ^2^ Department of Medical Oncology/Gastric Cancer Center, West China Hospital, Sichuan University, Chengdu, Sichuan, China

**Keywords:** DNA vaccine, immune checkpoint inhibitors, intramuscular gene delivery, pDNA, L/E/G

## Abstract

Immune check inhibitors (ICIs) have moderate response rates (~20%–30%) in some malignancies clinically, and, when used in combination with other immunotherapeutic strategies such as DNA tumor vaccines, there is evidence to suggest that they could optimize the efficacy of cancer treatment. In this study, we validated that intramuscular injection of plasmid DNA (pDNA) encoding OVA combined with pDNA encoding α-PD-1 (abbreviated as α-PD-1 in the following treatment groups) may enhance therapeutic efficacy by means of *in situ* gene delivery and enhanced muscle-specific potent promoter. Mice treated with pDNA-OVA or pDNA-α-PD-1 alone showed weak tumor inhibition in the MC38-OVA-bearing model. In comparison, the combined treatment of pDNA-OVA and pDNA-α-PD-1 resulted in superior tumor growth inhibition and a significantly improved survival rate of over 60% on day 45. In the B16-F10-OVA metastasis model, the addition of the DNA vaccine enhanced resistance to tumor metastasis and increased the populations of CD8^+^ T cells in blood and spleen. In conclusion, the current research shows that a combination of pDNA-encoded PD-1 antibody and DNA vaccine expressed *in vivo* is an efficient, safe, and economical strategy for tumor therapy.

## Introduction

An effective anti-tumor treatment requires not only the activation of immunity but also the inhibition of some inhibitory targets. Immunotherapy has attracted interest as a new method for cancer treatment. Blocking immune checkpoint molecules, including the CTLA4, PD-1, and PD-L1 molecules, was proposed as a highly effective anti-tumor treatment for most types of cancer (1, 2), with a 20%–50% success rate in different clinical trials (3). However, immune checkpoint inhibitor (ICI) therapy is still ineffective in most cancers, with the majority of cancer patients receiving little benefit from it. Nevertheless, its combination with other treatment methods is considered to be good treatment protocol and could have a tangible curative effect (4, 5).

In the growing field of immunotherapy, because of ability to promote extensive immune activation, DNA vaccines have good prospects as a treatment method for the prevention and cure of many cancers (6). Nevertheless, DNA vaccines have not received enough attention in clinical applications because of insufficient immune response as far. However, the application of new technologies has increased the immunogenicity of DNA vaccines; for instance, the optimization of plasmids and the utilization of gene delivery and genetic adjuvants has been improved through *in vivo* electrotransfer (7). The combined use of immune checkpoint inhibitors and DNA vaccines has a solid theoretical foundation. The transfection of plasmid DNA (pDNA) *in vivo* enables specific tissues to successfully produce desired proteins; pDNA can then either encode antigens or therapeutic proteins and is therefore a promising prospect in the treatment of various diseases. Recently, a number of studies have used the *in vivo* transfection of plasmids to produce therapeutic antibodies for tumor therapy (8–10).

To enhance this gene delivery efficiency, using an effective transport technique is of the utmost importance, as the phospholipid bilayer renders it impossible for any polar molecule, including DNA, to shuttle freely through the cell membrane. Numerous strategies have been explored to enhance the efficiency of penetrating cell membranes. In recent decades, researchers have made many efforts to boost the immunogenicity of DNA vaccines and gene delivery efficiency in bids to increase their clinical application potential. These strategies include the use of electroporation (EP) (7), which aims to increase the permeability of cell membranes and thus allow polar molecules to pass through.

Skeletal muscle is extensively distributed throughout the human body, and is both large in surface are and thick with blood vessels. To ensure that genes can be expressed at high levels in skeletal muscle without affecting the normal function of different tissues, we screened and developed muscle-specific promoters named efficient muscle-specific (EMS) promoters. By way of EMS promoters, the pDNA carrying the target gene is transferred to patien's skeletal muscle cells. The skeletal muscle cells function as “factories” that produce therapeutic proteins/polypeptides that are then secreted from cells, identifying their targets, and transported to them through blood vessels (11). A new finding related to the use of electroporation-mediated DNA shuttling is that several neo-antigens have been shown to effectively induce the anti-tumor function of CD8^+^ T cells in animals (12).

Here, we outline a method that utilizes DNA vectors as vaccine and immune checkpoint blockade (ICB) protein-encoding gene carriers. The pluronic L64 (L)/electroporation (E)/epigallocatechin gallate (G) (EGCG) (referred to as L/E/G) system was utilized to promote gene delivery efficiency. The aggregate of plasmid DNA encoding ovalbumin (OVA) (referred to as pOVA) vaccine with pVAX-α-PD-1 caused enormous tumor regression, which is often attributed to the elevated cytokine stage and proportion of CD8^+^ T and CD4^+^ T cells in tumors. We clearly demonstrate that this aggregate routine, via the L/E/G system, can prevent lung metastasis in B16F10-ovalbumin (OVA)-bearing mice models. In summary, we show that OVA vaccines with a modified delivery route can increase the efficacy of ICI, and that the plasmid encoding α-PD-1 provides a safer and more economical treatment choice for major cancer patients.

## Materials and methods

### Plasmids

The published complementary DNA (cDNA) sequences were used to construct the mouse PD-1 antibody (EP 1445 264 A1), and the PD-1 antibody sequence was cloned onto the pVAX1 vector. The OVA gene was cloned on the pcDNA3.1(+) and efficient muscle-specific plasmid (pEMS) to construct the DNA vaccine. The pEMS is a reconstructed plasmid backbone, replacing the cytomegalovirus (CMV) promoter of pcDNA3.1(+) with EMS (Patent CN 113106094 B). In accordance with the manufacturer’s instructions, all plasmids were obtained using an Endofree Plasmid Extraction Kit (Cwbio, Jiangsu, China) and dissolved in phosphate-buffered saline (PBS). The purities and concentrations of plasmids were determined using agarose gel electrophoresis and spectrophotometry.

### Cells

HEK293T, MC38, and B16F10 cells were purchased from American Type Culture Collection (ATCC, Manassas, VA, USA), and cultured in Dulbecco’s modified Eagle medium (DMEM) (Gibco, ThermoFisher, USA) containing 10% fetal bovine serum (FBS) (Sigma Aldrich, Taufkirchen, Germany) and 1% penicillin/streptomycin (Sigma Aldrich, Taufkirchen, Germany). MC38 cell line (MC38-OVA) and B16F10 cell line (B16F10-OVA) stably expressing ovalbumin were established by *Lentivirus* transfection. MC38-OVA and B16F10-OVA cells were cultured in the above-mentioned DMEM media and supplemented with 2 μg/mL puromycin at 37°C at a concentration of 5% CO_2_ to sustain the expression of OVA. All cell lines were routinely tested and confirmed as being free of *Mycoplasma* contamination.

### Mice

C57BL/6J 6- to 8-week-old male mice were purchased from Sipeifu Biotechnology Company (Beijing, China). The mice were fed in a pathogen free environment, and kept in a room with a light/dark cycle of 12 hours. All animal experiments were approved by the Biomedical Research Ethics Committee of West China Hospital in Sichuan Universit in accordance with the guidelines set out by the Association for Assessment and Accreditation of Laboratory Animal Care International (AAALAC). MC38-OVA cells (5 × 10^5^) resuspended in PBS were subcutaneously injected into C57BL/6 mice. In this experiment, the date of tumor inoculation was set as day 0. The tumor volume was measured once every 3 days with an electronic digital caliper, which calculated the volume as follows: (width^2^ × length)/2. Results were displayed with a Kaplan–Meier survival curve and tumor growth curve. The mice to which MC38-OVA tumors were attached were euthanized on the 14th day after tumor injection; after their death, tumor, blood, and spleen samples were collected for further analysis. The mice were euthanized when close to death or when their attached tumors were larger than 3,000 mm^3^ in size. To establish the lung metastasis model, B16F10-OVA (1 × 10^6^) cells resuspended in PBS were inoculated on C57BL/6 mice via the caudal vein. The remaining mice were euthanized on day 29, and the organs needed for the experiment were collected.

### L/E/G system

Intramuscular gene delivery was administrated using the previously established L/E/G system (13). Briefly, L/E/G refers to a combined pluronic L64/electroporation/EGCG system. Following this system, pDNA was dissolved in EGCG (E4143, Sigma-Aldrich, Germany) working solution and incubated for 30 minutes at room temperature. Subsequently, the EGCG/pDNA complex was mixed with 0.4% (W/V) L64 (435449, Sigma-Aldrich, German) until L64 reached a concentration of 0.1%. Finally, the EGCG/pDNA/L64 complex was injected into the right anterior tibial muscle of the mice; 1 hour later, the mice were stimulated with an electrical pulse to promote gene transfer.

### Immunoblotting analysis

Proteins in the supernatant that was collected from the transfected HEK293T cells were separated by 10% SDS-PAGE gel using 100 V for 2 hours and transferred to a polyvinylidene difluoride (PVDF) membrane. The membranes were blocked with 5% skimmed milk (RM00014, ABclonal, China) for 1 hour at room temperature and then incubated with HRP goat anti-mouse lgG (H+L) secondary antibodies (31430, ThermoFisher, USA), which can bind the Fc region of α-PD-1 expressed protein in mice, at a 1:5,000 dilution overnight at 4°C. The next day, the membranes were washed three times with tris-buffered saline with 0.1% Tween^®^ 20 detergent (TBST) and, subsequently, the protein bands were detected using enhanced chemiluminescence (ECL) with a ChemiDoc XRS machine and an image analyzer (Bio-Rad, USA).

### Immunohistochemical analysis

Immunostaining of lymphocytes in excised tumor tissues was carried out in accordance with the method described in a previous study (14). The paraffin sections were probed with primary anti-CD4 antibody (14–0042–82, ThermoFisher, USA) and anti-CD8 antibodies (14–0081–82, ThermoFisher, USA) mAb.

### Side effects study

Blood was collected and left to coagulate at room temperature for 30 minutes. The clotted blood was then centrifuged at 3,500 rpm for 15 minutes at room temperature. After the serum was collected and stored at –80°C, the frozen serum samples were shipped to Li lai Co. Ltd. (Chengdu, China) for aminotransferase (AST) and alanine aminotransferase (ALT) analysis. The weights of spleens, lungs, and tumors were measured on day 29.

### Enzyme-linked immunospot

The IFN-γ Single-Color enzyme-linked immunospot (ELISpot) assay was carried out on the mice samples in accordance with the manufacturer’s instructions (2210002, Dakewe Biotech, China). The procedure carried out was as follows: 1 × 10^5^ fresh splenocytes were diluted in 100 µL of CTL-Test medium (Dakewe Biotech, China) and incubated overnight at 37°C on an anti-IFNγ-coated 96-well plate. For stimulation, 10 ng/µL of TRP2_180–188_ peptide (SVYDFFVWL) (GenScript, Nanjing, China) was added to the plate and cultured for 2 days. A cell stimulation cocktail (CT317-PR2, Dakewe Biotech, China) was used as a positive control and PBS and a P815 irrelevant peptide (LPYLGWLVF) were used as negative controls. Finally, the spots were counted using an ELISpot reader system (Xingjianya, China).

### ELISA

For the quantitative determination of cytokine levels in serum, the blood of mice was collected from the orbital veins, then serum was separated at room temperature and stored at -80°C. The concentrations of interleukin-2 (IL-2) (pg/mL) (RK00007, ABclonal, China), IL-4 (pg/mL) (RK00036, ABclonal, China), and IL-10 (pg/mL) (RK00016, ABclonal, China) were determined using commercial enzyme-linked immunosorbent assay (ELISA) kits.

Binding ELISA was conducted to verify the affinity of antibody in the supernatant from pVAX-α-PD-1-transfected cells. The procedure carried out was as follows: 96-well plates were pre-coated with 10 μg/mL of PD-1 protein (Sigma) overnight at 4°C. They were subsequently washed with 0.1% Tween 20/PBS and then blocked with 5% bovine serum albumin (BSA) in a humid chamber for 1 hour at room temperature. After blocking, plates were washed three times and incubated at room temperature with supernatant from cells for 1 hour. Next, plates were washed and incubated with HRP goat anti-mouse lgG (H+L) secondary antibody at a dilution of 1:10,000 for 1 hour. The optical density was measured at 450 nm with an automatic ELISA reader (Synergy H1 Microplate Reader, BioTek, USA).

### Flow cytometry analysis

Peripheral blood and spleens were collected 14 days after tumor implantation. Spleens were pressed into single-cell through a 70 μm cell strainer (251200, Sorfa, China), followed by removal of the red blood cells with RBC lysis buffer (00430054, ThermoFisher, USA). Cells were incubated for 10 minutes on ice with anti-mouse CD16/32 antibody (14–0161–82, Thermo Fisher, USA) for Fc blocking. For CD4^+^ and CD8^+^ T-cell detection, cells were stained with eBioscience™ Fixable Viability Dye eFluor™ 450 (65–0863–14, Thermo Fisher, USA), anti-CD45 (63–0451–82, Super Bright™ 600 rat anti-mouse CD45, Thermo Fisher, USA), anti-CD3 (11–0031–82, FITC rat anti-mouse CD3, Thermo Fisher, USA), anti-CD4 (17–0042–82, APC rat anti-Mouse CD4, Thermo Fisher, USA), and anti-CD8 (45–0081–80, PerCP-Cyanine5.5 rat anti-mouse, Thermo Fisher, USA) antibodies for 30 minutes at 4°C. Blood samples were stained directly, then mixed with 1 mL of RBC lysis buffer and incubated at 37°C for 5 minutes so that red blood cells could be lysed. Sample data were acquired with LSRFortessa (BD bioscience, NJ, USA) and analyzed with FlowJo software (FlowJo, Ashland, OR, USA).

### Statistical analysis

Statistical analyses were carried out using GraphPad Prism software for Windows. For all experiments, statistical significance was determined using one-way analysis of variance (ANOVA) or a Student’s *t-*test. The survival rate of mice was calculated using the log-rank test. In all cases, differences between groups were considered significant when the *p*-value was < 0.05 (*p < 0.05, **p < 0.01, and ***p < 0.001); in some cases there was no significant difference (NS).

## Results

### pVAX-α-PD-1-Fc can express antibodies with mouse PD-binding activity *in vitro*

**Figure 1:**
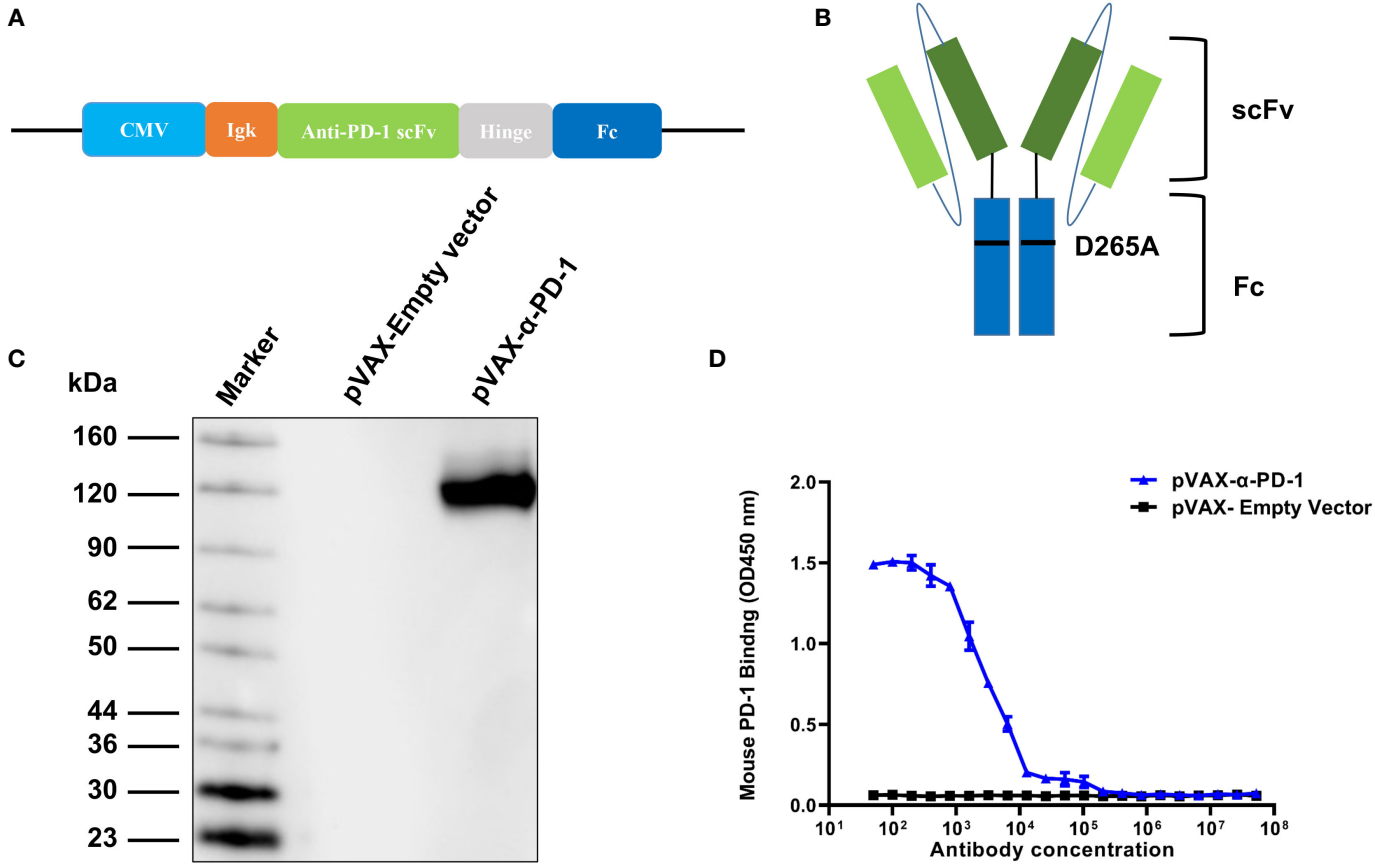
Expression and binding of PD-1 antibody. **(A)** Schematic of DNA construct encoding PD-1 antibody. The α- PD-1-Fc gene was cloned onto the pVAX vector and driven by the CMV promoter. **(B)** Structure of PD-1 antibody. α-PD-1-Fc contained scFv of mouse PD-1 antibody and the Fc region of mouse IgG1 with a D265A mutation. **(C)** Western blot analysis of mouse IgG from supernatants of pVAX-α-PD-1-Fc transfected 293T under non-reduced conditions. The pVAX-empty vector served as a control. Numbers indicate molecular weight. **(D)** The binding ELISA of α-PD-1-Fc from cell culture supernatants with mouse PD-1 antibodies. Results are representative means ± SDs of three independent experiments.

A DNA molecule capable of directing *in vivo* antibody production was designed by creating a cassette consisting of the single-chain variable fragment of anti-mouse PD-1(J43 clone) and the mouse Fc region with a D265A point mutation which leads to the total loss of cytolytic function and cloned onto pVAX plasmid (**Figures 1A, B**). To ensure that the plasmid production of PD-1 antibody, human 293T cells were transfected with pVAX and pVAX-α-PD-1-Fc. In the culture media of cells transfected with pVAX-α-PD-1-Fc, western blot assay under non-reducing conditions detected secretion of 120-KDa protein (**Figure 1C**). The binding affinity of α-PD-1 to the corresponding mouse antigen PD-1 was detected using an ELISA assay. The ELISA assay showed that α-PD-1 from cell culture media bound to PD-1 protein in a dose-dependent manner and that it had a significantly higher binding capacity than the control group (**Figure 1D**). The results of the assay also demonstrated that pVAX-α-PD-1 was capable of encoding functional mouse PD-1 antibodies.

### pEMS-OVA vaccine combined with PD-1 antibody expressed by plasmid delivery intramuscularly *in vivo* increased the anti-tumor activity in MC38-OVA model

**Figure 2:**
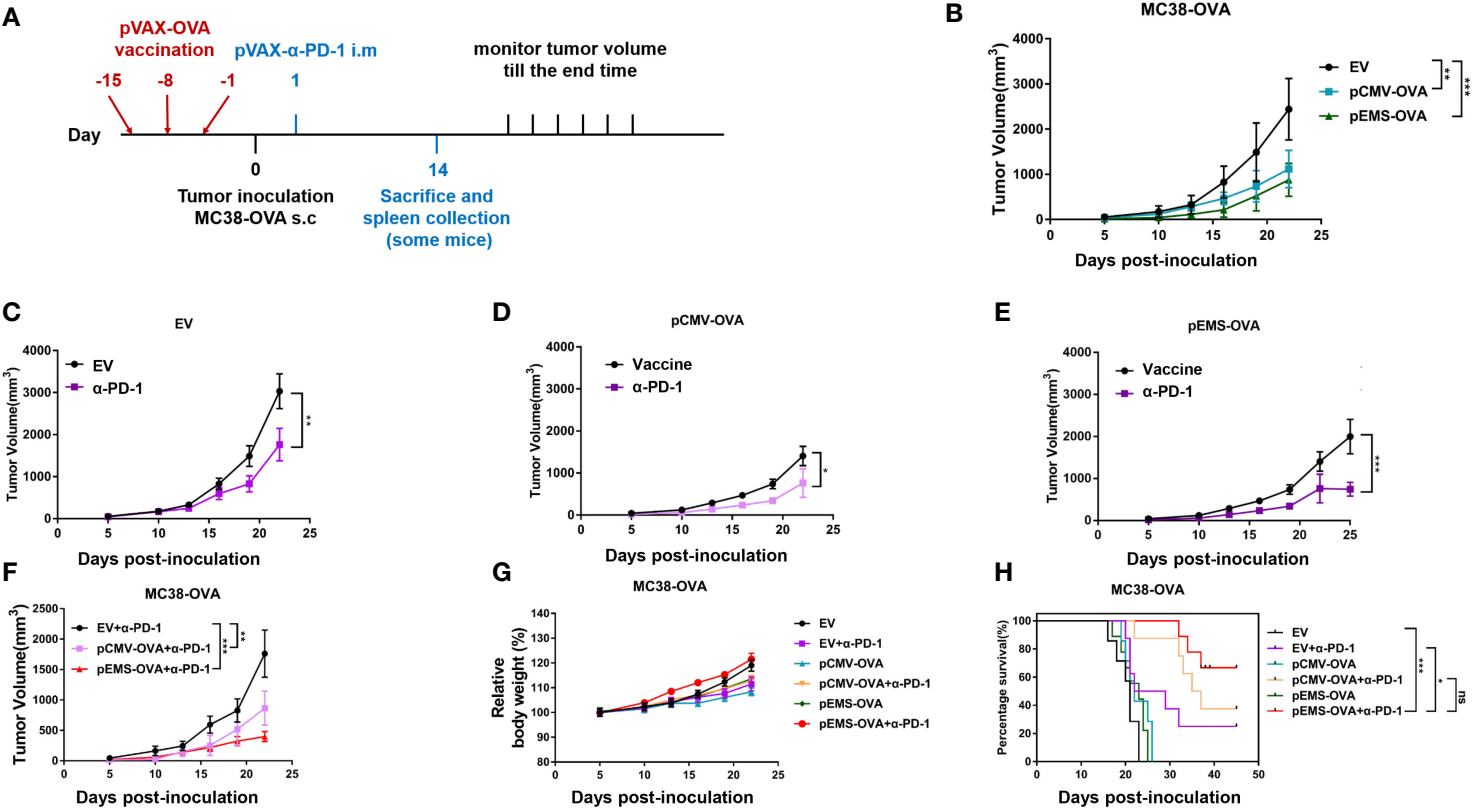
Anti-tumor activity of DNA vaccination and intramuscular gene transfer of PD-1 antibody combination in MC38-OVA model. **(A)** Schematic protocol of the tumor inoculation, DNA vaccination and pVAX-α-PD-1-Fc injection, and tumor measurement in MC38-OVA model. The DNA vaccines (pCMV-OVA or pEMS-OVA) were pre-intramuscularly electroporated 15 days, 8 days, and 1 day before 2 × 10^5^ MC38-OVA tumor cells were subcutaneously injected into the right flanks of C57BL/6 mice. pVAX-α-PD-1-Fc were intramuscularly electroporated on day 1. Thereafter, the tumor volume was monitored every 3 days until mice were sacrificed. **(B)** Tumor growth curves of three different vaccine groups. The tumor growth curves of the empty vector **(C)**, pCMV-OVA **(D)**, and pEMS-OVA **(E)** DNA vaccination combined with pVAX-α-PD-1-Fc groups. **(F)** Tumor growth curves of three kinds in the pVAX-α-PD-1-Fc group. Relative body weight change **(G)** and percentage of survival curve **(H)** of mice receiving six different treatments. The error bars represent the mean ± SEM; *n* = 6 per group. Statistical analysis was carried out using one-way ANOVA with Dunnett’s multiple comparisons test or a Student’s *t*-test. The log-rank (Mantel-Cox) test was used to calculate the significance of survival (*p < 0.05, **p < 0.01, and ***p < 0.001). ANOVA, analysis of variance.

We examined the tumor inhibition effect induced by the pCMV/EMS-OVA DNA vaccine combined with pVAX-α-PD-1 on the tumor progression in MC38-OVA-bearing mice. The mice were divided into three groups according to treatment type, i.e., the pCMV-empty vector, pCMV-OVA, and pEMS-OVA groups. These groups were evenly divided into two groups after tumor formation, and they were treated with pVAX-Ctrl or pVAX-α-PD-1, respectively. DNA vaccine injections, tumor inoculation, and pVAX-α-PD-1 injections were administered on different days (**Figure 2A**), and we examined the effects of these different vaccination pathways on tumor sizes. The pCMV-OVA vaccination showed little tumor inhibition effect, and the pEMS-OVA vaccination caused the greatest delay in tumor growth (**Figure 2B**). All mice that received the pVAX-α-PD-1 treatment displayed slower tumor growth than those administered with DNA-only vaccine treatment (**Figures 2C–E**). In particular, mice that were in the pEMS-OVA combined with pVAX-α-PD-1 treatment group showed significant tumor regression (**Figure 2F**). For safety reasons, we monitored the mice for changes in body weight after tumor inoculation. All mice, irrespective of group, displayed stable weight gain (**Figure 2G**), and this preliminarily finding indicated that these treatments were safe enough. However, mouse survival rate varied significantly across the groups, and all mice in the pCMV-empty vector combined with pVAX-Ctrl treatment group (EV) died within 23 days of tumor inoculation. The pEMS-OVA vaccine combined with pVAX-α-PD-1 treatment group (pEMS-OVA + p-α-PD-1) showed superior results, with about 60% of the mice surviving for more than 45 days. By contrast, approximately 40% of the mice in the pCMV OVA vaccine combined with pVAX-α-PD-1 treatment group (pCMV-OVA + p-α-PD-1) survived for more than 45 days and approximately 20% of the mice in the pVAX-α-PD-1 single treatment group (EV + p-α-PD-1) survived for more than 45 days (**Figure 2H**). The results above demonstrate that the pEMS-OVA vaccine combined with pVAX-α-PD-1 treatment provided the greatest therapeutic benefit.

### DNA vaccination in combination with PD-1 antibody expressed by intramuscular plasmid transfer enhanced the immune response of MC38-OVA-bearing mice

**Figure 3:**
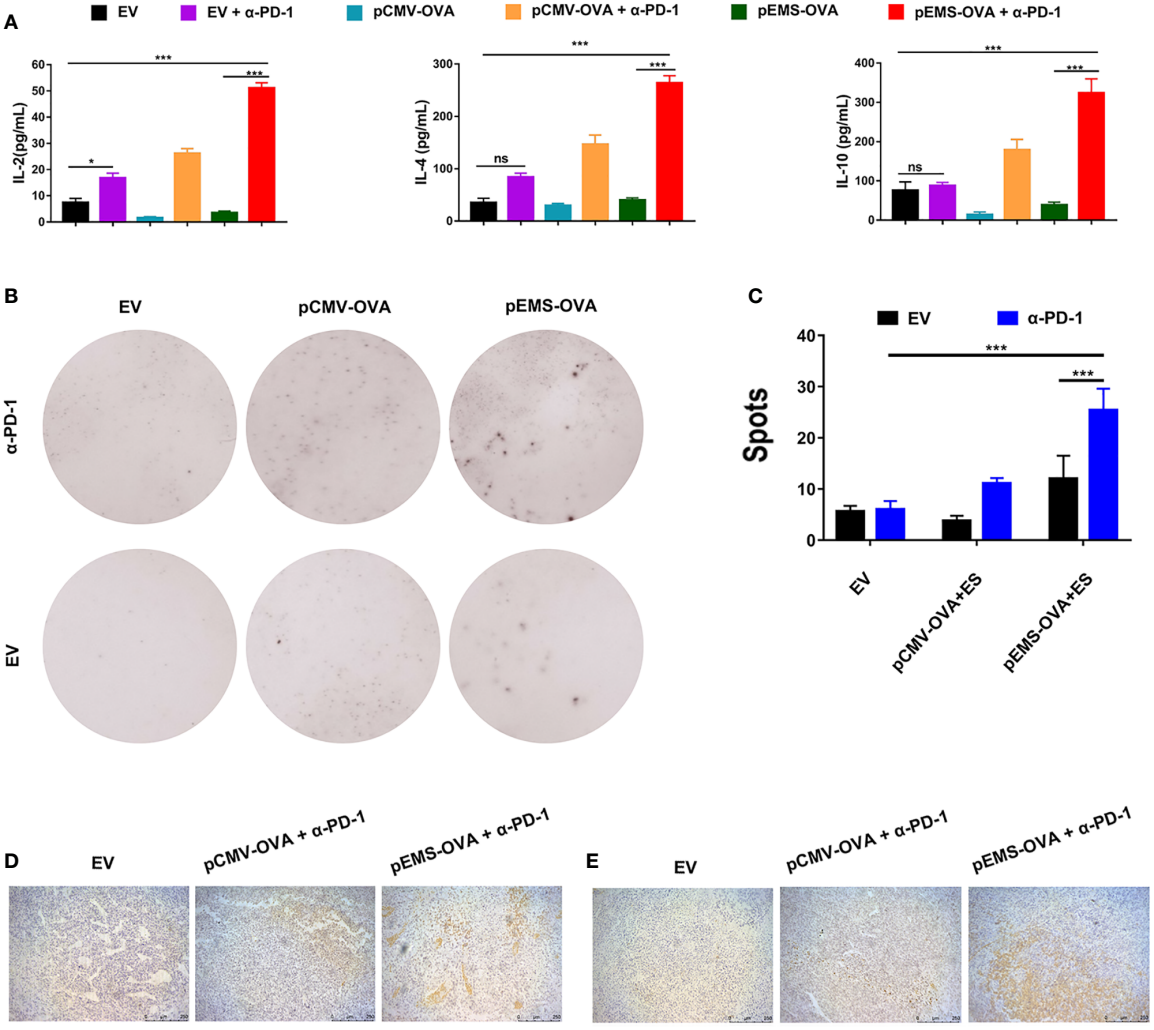
Immune response effect of DNA vaccination and intramuscular gene transfer of PD-1 antibody combination for MC38-OVA-bearing mice. **(A)** Levels of IL-2 (left), IL-4 (middle), and IL-10 (right) inflammatory mediators in the serum of mice receiving six different treatments were detected by ELISA on day 29. **(B)** Representative IFN-γ ELISPOT wells for splenocytes isolated from various six treatment groups. **(C)** Quantification of IFN-γ positive spots in the six treatment groups. Immunohistochemical analysis of CD4^+^ **(D)** and CD8^+^ **(E)** T-cell infiltration in MC38 tumors from mice treated with three kinds of vaccine combined pVAX-α-PD-1-Fc groups. Scale bars: 250 μm. Results are representative of three independent experiments and are expressed as the mean ± SD; *n* = 5 per group. One-way ANOVA was used to calculate the *p*-value, *p < 0.05, ***p < 0.001. ANOVA, analysis of variance; ELISPOT, enzyme-linked immunospot.

To study the activation levels of positive response in tumor microenvironment (TME) brought about by a combination of vaccines and pVAX-α-PD-1 therapy, the concentrations of IL-2, IL-4, and IL-10 cytokines in the serum were determined. The concentrations of IL-2, IL-4, and IL-10 were at very low levels in the vaccine-only treatment groups (i.e., the pCMV-empty vector, pCMV-OVA and pEMS-OVA groups). However, the concentration of IL-2 cytokines was slightly increased in the pCMV-empty combined with pVAX-α-PD-1 treatment group. Meanwhile, the concentrations of IL-2, IL-4, and IL-10 cytokines were significantly increased in the serum after the DNA vaccine combined with pVAX-α-PD-1 treatment, indicating that responses that were not triggered by DNA vaccine had been elicited (**Figure 3A**). We further evaluated the capacity of the DNA vaccine combined pVAX-α-PD-1 treatment to elicit a particular IFN-γ response by ELISpot. Without the vaccine, very few spots were detected, but the splenocytes from the DNA vaccine-treated mice showed a significant increase in the number of spots. In addition, the stimulation of immune cells with the DNA vaccine combined with pVAX–α-PD-1 treatment resulted in a significantly increased number of spots being observed (**Figures 3B, C**). To investigate whether the EMS promoter is more beneficial for immune activation after intramuscular injection of plasmids. In the next experiments, the mice were limited to three main combination groups that revealed significant efficacy after treatment. Fifteen days after tumor induction, MC38-OVA-bearing mice were euthanized and tumors were excised. The levels of CD8^+^ T and CD4^+^ T cells were higher in the tumor tissues of the therapeutic groups than those of the empty vector group (**Figures 3D, E**).

These results showed that the pEMS-OVA vaccine combined with pVAX-α-PD-1 treatment activated more antigen-specific lymphocyte cells than the pVAX-α-PD-1 treatment alone. In addition, increased PD-1 expression in cytotoxic T lymphocyte (CTL) indicated T-cell exhaustion and the loss of effector function (15, 16), and led to tumor growth. These results indicate that the combined pEMS-OVA vaccine and pVAX-α-PD-1 treatment leads to the increased recruitment of CTL into tumors, and thus has a significant anti-tumor effect.

### DNA vaccinations potentiated the inhibition of melanoma pulmonary metastases by intramuscular pDNA-based PD-antibody

**Figure 4:**
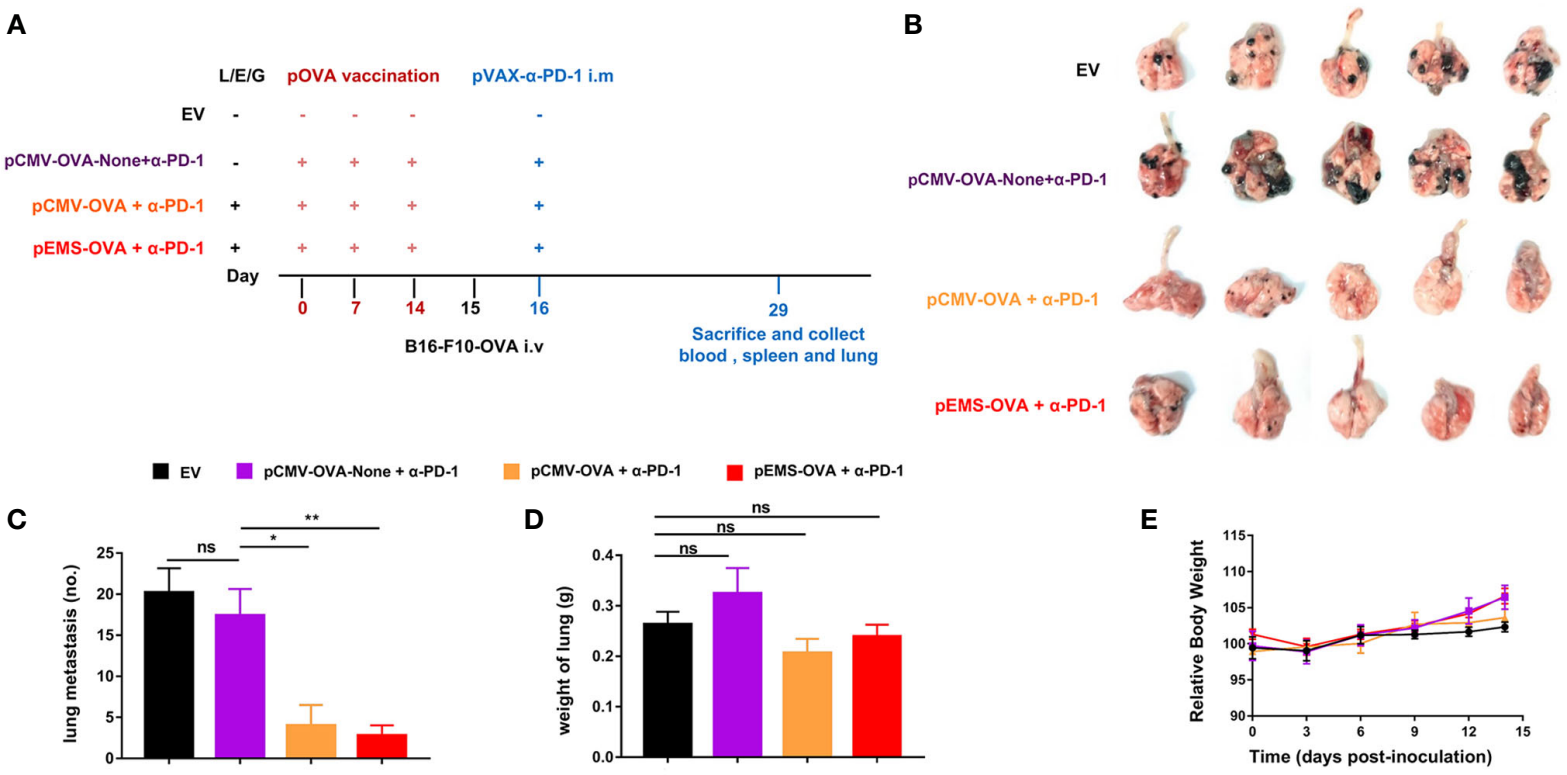
Lung metastasis inhibition of combination DNA vaccination and intramuscular gene transfer of PD-1 antibody in B16F10-OVA model. **(A)** Schematic protocol of the B16F10-OVA inoculation and combination therapy of DNA vaccination with pVAX-α-PD-1-Fc. C57BL/6 mice were divided into four groups: the combination of pOVA vaccine with α-PD-1 antibody (pCMV-OVA + α-PD-1 and pEMS-OVA + α-PD-1), the combination of vaccine without L/E/G and α-PD-1 (pCMV-OVA-none + α-PD-1) and no vaccine (empty vector). *N* = 5 per group. **(B)** Images of lung metastasis taken 2 weeks after B16F10-OVA inoculation. Number of metastases nodules on the surface of lung **(C)** and the wet weight of lungs **(D)** of the four groups. **(E)** Relative body weight change of mice receiving four different treatments. The data **(C–E)** represent the means ± SDs and were subjected to a one-way ANOVA; “NS” indicates no significant difference. *p < 0.05; **p < 0.01. ANOVA, analysis of variance; pluronic L64 (L)/electroporation **(E)**/epigallocatechin gallate **(G)** (EGCG) (referred to as L/E/G).

Because cancer cells can spread from primitive tumors to different parts of the body even in the early stages, the inhibition of metastasis is an essential attribute of cancer vaccines. We tried to determine if pOVA vaccines (i.e., pCMV-OVA and pEMS-OVA) could enhance the inhibitory effect of intramuscular pDNA-based PD-1 antibody using a poorly immunogenic B16-F10 lung metastases model. We used the same treatment protocol as for the MC38-OVA model (**Figure 4A**). The weakest inhibitory effect on lung metastasis was observed in the empty vector treatment group. We also discovered the importance of the L/E/G system to vaccination, as we observed that, as in the empty vector group, there was weak suppression of lung metastasis in the DNA vaccination without L/E/G (pCMV-OVA-None + p-α-PD-1) treatment group. The pCMV-OVA combined with pVAX-α-PD-1 (pCMV-OVA + p-α-PD-1) therapy strongly suppressed the lung metastasis of tumors, which was consistent with findings in previous studies (17–19). The effect of α-PD-1 therapy is mainly dependent on innate immunity and the infiltration of lymphocytes in tumors (20, 21). We observed that pEMS-OVA combined with pVAX-α-PD-1 (pEMS-OVA + p-α-PD-1) therapy further prevents the formation of pulmonary nodules (**Figures 4B, C**).

**Figure 5:**
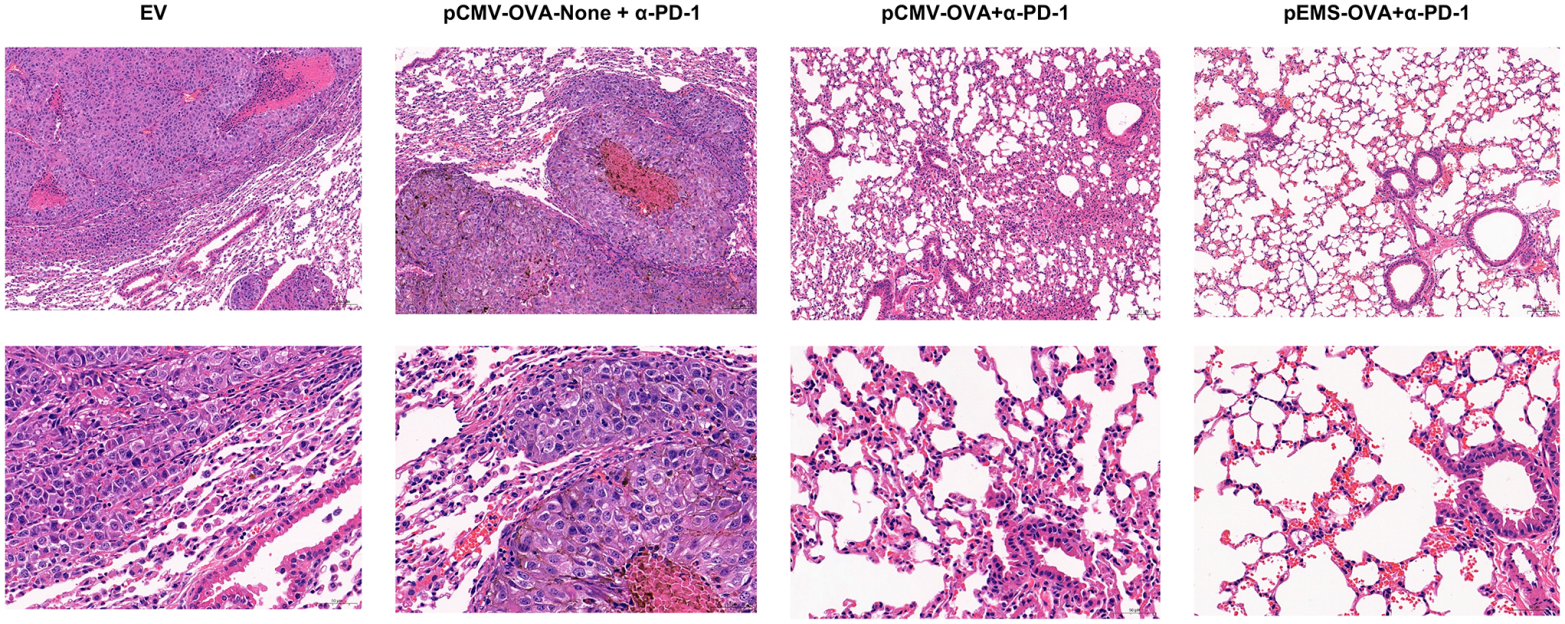
H&E staining of lungs collected from the treated B16F10-OVA-bearing mice. Representative H&E staining images of lungs isolated from B16F10-OVA-bearing mice following treatment with a combination of different DNA vaccination and intramuscular gene transfer of PD-1 antibody. H& E staining, hematoxylin and eosin staining.

**Figure 6:**
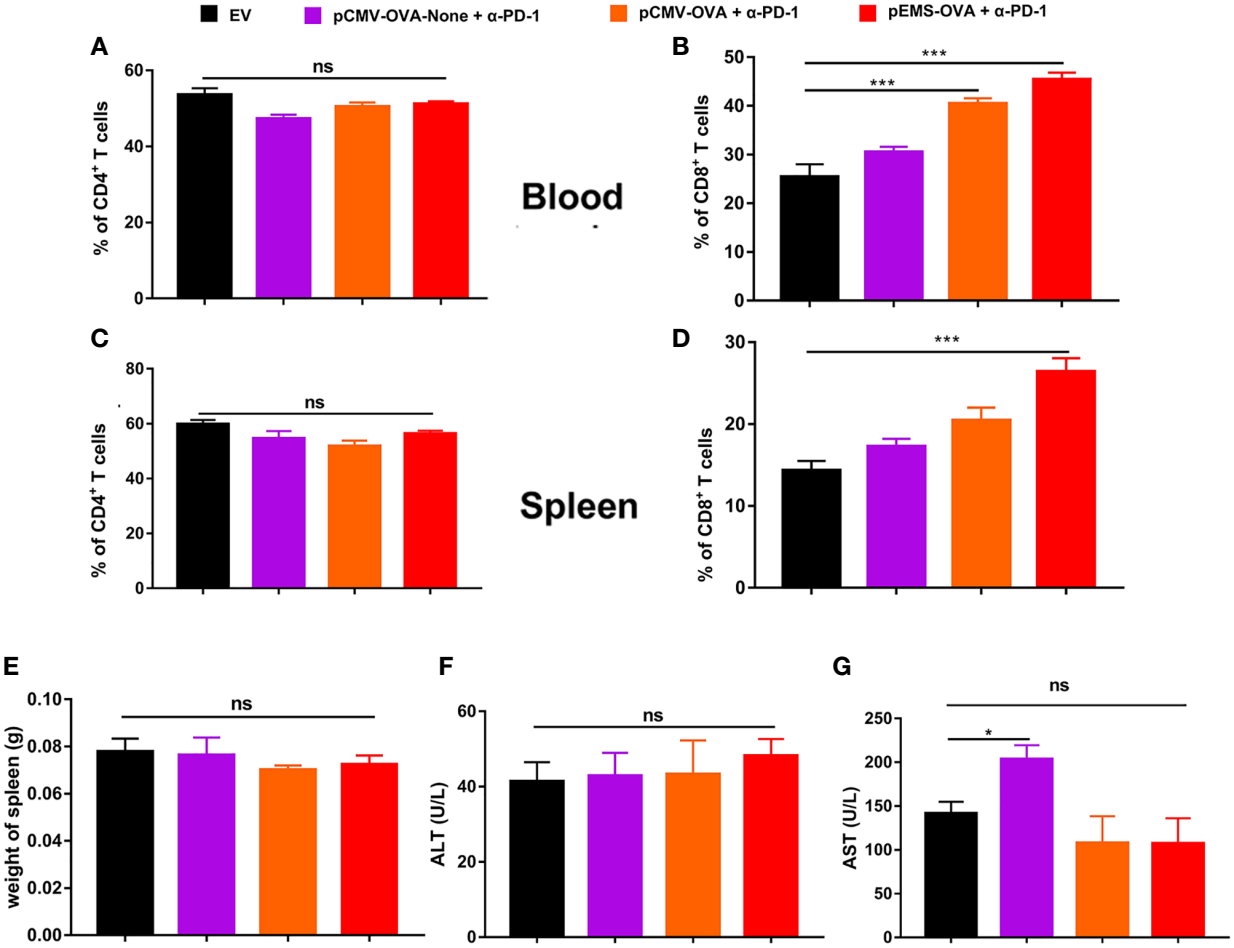
Immune cell populations and biochemical analysis of combination DNA vaccination and intramuscular gene transfer of PD-1 antibody in B16F10-OVA model. All mice were sacrificed at day 29. Immune cell populations analysis of spleen and blood from mice receiving four different treatments. **(A)** Percentage of CD4^+^ T cells in the blood. **(B)** Percentage of CD8^+^ T cells in the blood. **(C)** Percentage of CD4^+^ T cells in the spleen. **(D)** Percentage of CD8^+^ T cells in the spleen. **(E)** Spleen weight. **(F)** ALT level in the serum of mice. **(G)** AST level. Data are shown as means ± SDs. Statistical analyses were performed using one-way ANOVA with Dunnett’s multiple comparison test. *p < 0.05; **p < 0.01; ***p < 0.001. ANOVA, analysis of variance; AST, aminotransferase; ALT, alanine aminotransferase. .

In excised lungs, a trend for increased lung weight was observed in the DNA vaccination without L/E/G (pCMV-OVA-None + p-α-PD-1) treatment group, although there was no statistical difference in comparison to the other groups (**Figure 4D**). Similarly, the hemoxylin and eosin (H&E) staining of lung tissues identified intensive metastases in control mice and mice in the DNA vaccination without L/E/G treatment group, whereas there were notably small numbers of nodules detected on the lung sections of mice in the pCMV-OVA combined with pVAX-α-PD-1 and the pEMS-OVA combined with pVAX-α-PD-1 treatment groups. Remarkably, the combination of pVAX-α-PD-1 and pEMS-OVA vaccine therapy brought about almost normal lung histopathology (**Figure 5**), and this efficacy may be attributed to increased CD8^+^ T-cell ratios in the blood and spleen (**Figures 6B, D**). There was no demonstrable difference, however, in the proportion of CD4^+^ T cells, both in the blood and spleen, across the treatment groups (**Figures 6A, C**). However, in the pEMS-OVA combined with pVAX-α-PD-1 treatment group, the proportion of CD8^+^ T cells was increased by up to 45%, and the proportion in the spleen was increased by up to 27%. The proportion of CD8^+^ T cells in the spleen and peripheral blood were also significantly elevated (**Figures 6B, D**).

Finally, we evaluated the safety of the treatments. The results showed that there was no increase in lung water content or splenomegaly after any treatment (**Figures 4D**, **6E**). Moreover, mouse body weight increased slightly during the treatment course (**Figure 4E**). No significant difference was detected in ALT levels between the four groups (**Figure 6F**). However, AST levels were increased in the serum of the pCMV-OVA-none combined with pVAX-α-PD-1 treatment group when compared with the empty vector group, and this may be related to the malignant metastasis activity of the tumors. However, in the sera of the two vaccines with combined PD-1 antibody treatment groups, we observed decreased AST levels when compared with the empty vector group, but these differences were not significant (**Figure 6G**).

Overall, our data indicate that the pEMS-OVA combined with pVAX-α-PD-1 treatment had no apparent serious side effects and had enhanced anti-tumor effects. Potential mechanisms for synergistic effects of DNA vaccine may be attributed to the vaccine’s ability to boost CD8^+^ T cells, the exhaustion of which may have been prevented by the inhibition activity of PD-1 antibodies, expressed *in vivo*. 

## Discussion

Immune checkpoint inhibitors, notably antibodies targeting PD-1 and CTLA4, have revolutionized the treatment strategies for many cancers, especially advanced melanoma, for which, currently, nearly 50% of tumors in patients can either regress or be controlled for long periods, compared to the historical figure of less than 10% (22). But achieving this ideal response with this treatment strategy is still difficult for many tumors. Recently, antibodies targeting PD-1, including pembrolizumab and nivolumab, have been approved as a second-line treatment plan for HCC patients. However, their objective response rates (ORR), being 20% and 16.9% for nivolumab and pembrolizumab, respectively, are not satisfactory (23, 24). This indicates that there are numerous patients with limited response to immunomonotherapy, and this has promoted the exploration of new tumor treatment strategies that may improve the effectiveness of immunotherapy for these unresponsive tumors.

Although satisfactory results have been obtained in preclinical models, the low immunogenicity of DNA vaccines and the adverse reactions of many patients to the blocking of immune checkpoints still limit their application in clinical environments. Previous studies have demonstrated that the intramuscular delivery of DNA is a promising therapeutic method. Because of its large therapeutic area, its accessibility, and the presence of many antigen-presenting cells within it, skeletal muscle was selected as an appropriate target, especially for the delivery of DNA plasmids encoding a variety of tumor antigens or immune checkpoint inhibitory antibodies. To make gene transfer by this means efficient, a variety of measures have been exploited to increase transfection efficiency (25).

Gene electrotransfer is a mature non-viral delivery strategy that has been used to deliver naked DNA or RNA to various types of tissue. As a commonly used delivery system, lipid nanoparticles (LNPs) provide support for the delivery and site-specific release of mRNA and DNA (26). An mRNA vaccine prepared with LNPs as carriers can therefore better resist the degradation of nuclease, making intravenous injection and other delivery routes possible. However, the clinical application potential of cationic liposomes is limited by their toxicity, non-specific immunogenicity, circulatory instability, and rapid clearance *in vivo* (26, 27). If LNPs are to be used more widely in clinical settings, more in-depth research is needed to solve the problems of biological distribution, insufficient immune cell recruitment, and clearance *in vivo* in target tissues (28, 29). mRNA is rapidly translated in the cytoplasm. The expression of therapeutic antibodies based on delivered RNA is more transient compared with that based on DNA or viral vector-mediated gene transfer (30). Although viral vectors can promote high levels of expression in the long term, it is difficult to repeatedly administer them and they may produce genetic markers in patients, and more attention ought be directed toward potential concerns with the safety of their use (31).

The L/E/G system has not been previously applied in the field of vaccine preparation. Under normal conditions, its components cause an immune reaction at the injection site and recruit cells such as macrophages, so it may be particularly suited to the production of vaccines. In this study, we used the L/E/G system to improve the transfer efficiency of plasmids. It can help plasmids to enter the nucleus efficiently. Traditional DNA vaccines must first be successfully transcribed in the nucleus before entering the cytoplasm to start the expression of antigen proteins. The nucleus's multi-layer barrier makes it difficult for DNA to enter the reaction site and therefore makes immune activation difficult. In this study, the L/E/G system could disturb the cell membrane and help plasmids to enter the muscle cells. In addition, it could deform pDNA so that it had a suitable architecture with an appropriate surface charge, prevent the degradation of nucleic acids, and improve delivery efficiency, thereby increasing the expression levels of exogenous genes in skeletal muscle cells (13). Using EMS promoter, OVA can be efficiently expressed in muscle, which is conducive to enhancing immune activation, thereby overcoming the obstacle of immune activation. Compared with the pathological alteration caused by the accumulation of lipids in liver of LNPs, L/E/G/does not cause pathological changes to main organs. Although the L/E/G system used here cannot overcome the relevant shortcomings of LNP, we still showed that it represents an available, economical, and efficient alternative to existing methods of gene delivery.

In this study, we explored the use DNA vaccine or plasmid encoded antibodies and the combined treatment of α-PD-1 with DNA vaccines through the L/E/G system, and found that the pEMS-OVA DNA vaccine can not only can prevent tumor attacks from occuring in the body, but also cooperate with pVAX-α-PD-1 plasmid to suppress tumors.

Based on studies related to neoantigen vaccination and immunomodulatory therapy, this study selected MC38 to construct a colon tumor model. It has been established that MC38 cells trigger spontaneous CD8^+^ T cell-induced immune responses in tumor-bearing mice. However, because of their immunosuppressive microenvironment, these immune cells are inactive and cannot eradicate tumor cells completely (32). In the early stages of tumor progression, the treatment of tumor-bearing animals with immunomodulatory antibodies targeting PD-L1 or PD-1 triggers effector T-cell responses, which can induce tumor cell death (33). In this study, we revealed that pOVA vaccines, in combination with α-PD-1 expressed by pVAX-α-PD-1, can trigger tumor death in a CD8^+^ T cell-dependent manner, and that treatment with α-PD-1 antibody alone moderately inhibited tumor growth.

In this study we also used a murine colon cancer model to determine what factors affected immune checkpoint inhibitor blockade, and, even though the vaccine vectors used were different, we observed that tumors were smaller in mice treated with pVAX-α-PD-1 than in those in the pVAX-empty vector group 15 days after tumor inoculation, which means that pVAX-α-PD-1 monotherapy can suppress tumor growth. In addition, tumor growth and the survival rate were substantially improved by pVAX-α-PD-1 treatment in the following experiment. Furthermore, when we examined different methods of immunization, we discovered that the pEMS-OVA expressed vaccine was associated with the highest tumor-suppressing efficiency. Our most important finding, however, was that the pEMS-OVA combined with pVAX-α-PD-1 treatment ameliorated the survival rate and reduced tumor growth. This synergistic effect originated from the ability of DNA vaccines and ICBs to stimulate T cells to fight specific cancer antigens, thereby increasing T-cell activity and tumor infiltration. Therefore, ICB creates a good microenvironment for the activity of cancer DNA vaccines. The pVAX-α-PD-1 treatment was found to significantly increase the quantity of IFN-γ-secreting CD8^+^ T cells. When used in combination, pOVA and pVAX-α-PD-1 not only increased levels of IL-2, IL-4, IL-10, and IFN-γ production but also demonstrably increased those of CD8^+^ and CD4^+^ T cells. IL-2 can not only adjust tolerance of the dysregulated immune system through regulatory T cells (Tregs) but also promote immune response through activating effector T cells and memory T cells (34). In early studies, IL-2 was co-administered with autologous lymphocytes for cancer treatment or for the amplification of patients’ natural killer (NK) cell and effector T-cell populations (35, 36). The combination of immunostimulatory plasmid DNA encoding IL-2 and siRNA delivered by TT-LDCP NPs targeting PD-L1 in the treatment of hepatocellular carcinoma (HCC) has also been shown to significantly increase tumor infiltration and CD8^+^ T-cell activation (37). The significant increase in the levels of IL-2 in the pEMS-OVA+α-PD-1 group may also indicate the up-regulated infiltration of CD8^+^ T cells. IL-4 is a Th2 cytokine that has a wide range of biological effects, especially with regard to immune responses (38). In the treatment of B-ALL with CD19/CD22/CD3 trispecific antibody, IL-4 was up-regulated in the treatment group (39). Another study showed that Man-CTS-TCL NPs increased the concentration of IL-4 concentration compared with the control group (40). After OVA stimulation, IL-4 levels in the spleen cells of mice increased significantly (41). Earlier studies have mainly focused on the immunosuppressive role of IL-10, but recent studies have shown that IL-10 can also reprogram the metabolic profile of T cells and restore the function of depleted T cells to produce a response (42). The tumor-triggered amplification of Tregs is an obvious obstacle to immunotherapy. Strikingly, however, AV-IL-27 treatment has been shown to induce the rapid elimination of Tregs in lymphoid organs and peripheral blood. AAV-IL-27 therapy also leads to tumor regression without significant adverse events, partly because of its induction of IL-10 (43). Further research has shown that IL-10 promotes the preservation of the effector function of antitumor CD8^+^ T cells (44). Interestingly, PD-1 blockade results in the compensatory release of IL-10 through tumor-infiltrating myeloid dendritic cells (45). Increased levels of IL-2/IL-4/IL-10 cytokine secretion after PD-1 antibody expressed by pVAX-α-PD-1 treatment were also observed in our study. The increased levels of IL-10 secretion in combination treatment groups may function through the the mechanism of compensatory IL-10 secretion by dendritic cells after PD-1 blockade. The production of cytokines is only one of many factors that affects tumor volume, so it is difficult to ensure that changes in tumor volumes are completely attributable to these observed trends. Immune activation starts to occur in the early phases of tumor development, especially when therapy combining DNA vaccines and ICBs is used.

In B16F10-OVA lung metastasis mice models, the combined use of ICBs and pOVA vaccines allowed us to reduce the doses of antibodies and induced a potentially more powerful immune response when compared with single therapy. The early stages of tumor development, that is up to 14 days after tumor injection, have been proven to be key to metastasis, and we therefore analyzed their role in the TME. The efficacy that suppresion of pulmonary metastases was benefit by an increase in the proportion of CD8. Consistent with these blood results, the combined use of ICBs and pOVA DNA vaccines can increase the percentage of CD8^+^ T cells in the spleen. In addition, pVAX-α-PD-1 without L/E/G treatment failed to inhibit lung metastases, indicating that the L/E/G system established in our previous study is sufficient to promote target gene expression and subsequent effects (13).

It is now clear that if a balance can be established between immune activation and immunosuppression, it is possible to achieve an effective anti-tumor response (46, 47) Therefore, the disadvantages of DNA vaccine or ICBs injection alone can be overcome by combining multiple treatment methods (48). In the case of cell vaccines, numerous in-depth studies have been conducted on the combined use of ICB and cancer vaccines (49, 50). Cell vaccine studies using triple therapy found that it is significantly superior to any dual or single therapies (49, 51). Strong anti-tumor effects were observed after treatment with a combination of cell-based vaccines and inhibitors. These can be attributed to antigen-specific effects, the increased proliferation of CD4^+^ T and CD8^+^ T cells, the release of antigen-specific cytokines, and the up-regulation of pivotal signaling molecules crucial to T-cell function (49). In contrast, few preclinical studies have tested the efficacy of DNA vaccines combined with immune checkpoint inhibitors (52). Published studies have explored the combination of single or dual CTLA4/PD-1 inhibitors and MYB oncoprotein targeting DNA vaccines (53), SSX2 cancer antigen (54), PSMA prostate-specific antigen (55), and TRP-2 and gp100 melanoma-specific antigens (56). The common characteristic of these studies is that combination therapy had better anti-tumor effects than single therapy. Some previous studies have demonstrated the feasibility of combined treatment of vaccines with ICI (57–60). As we know, the ICBs used in the above studies are antibodies (52, 61, 62), the costs of which are high, and the families of most cancer patients therefore cannot consider them as a treatment option. pVAX-α-PD-1 in this study utilized muscle cells as the production base of therapeutic antibodies. Currently, our research does not allow for the regulated expression of genes *in vivo*, and exploring gene switch platforms that control functional gene expression *in vivo* to enable self-regulatory therapy related to disease progression may provide a more valuable complement to this strategy (63). In addition, we can adjust the dosage of antibodies by changing the number of plasmids injected. Whether or not the effectiveness of this combined strategy is equally applicable to other tumor vaccines requires further study. The success of this strategy in preclinical research could make it possible for cancer patients to use cheap and affordable antibody drugs.

Our study reports on a specific combined treatment method. Driven by the muscle-specific promoter EMS, therapeutic proteins were specifically expressed in skeletal muscle to improve the effect of the vaccine. These observations show that the PD-1 antibody produced by intramuscular plasmid injection can synergize with DNA vaccines, most probably because of the highly increased effector function by expanding the frequencies of tumor-specific T cells. This combined strategy could represent a simple, convenient, economical, and effective means of oncology treatment.

## Data availability statement

The original contributions presented in the study are included in the article/supplementary material. Further inquiries can be directed to the corresponding authors.

## Ethics statement

The animal study was reviewed and approved by the West China Hospital of Sichuan University Biomedical Research Ethics Committee.

## Author contributions

XL and YY performed the experiments, analyzed data, and wrote the manuscript. GW and ML were involved in study conception and design. XZ was involved in data analysis and contributed intellectually to the research. All authors contributed to manuscript editing. All authors contributed to the article and approved the submitted version.
